# Flexible Low-Loss Thin Flimsy Stripline for High-Speed Connections

**DOI:** 10.3390/mi13122218

**Published:** 2022-12-14

**Authors:** Jau-Jr Lin, Yi-Da Tsai

**Affiliations:** Department of Electrical Engineering, National Changhua University of Education, Changhua 500, Taiwan

**Keywords:** PTFE, stripline, transmission line, MIMO, antenna array

## Abstract

Increasing numbers of antennas are being placed inside laptop screen bezels. Connections between antennas and laptop bases have become challenging owing to space limitations. Thus, this paper proposes a flexible low-loss thin flimsy stripline structure for high-speed applications. The cable should be sufficiently thin to avoid causing a water ripple effect while under the screen panel. Furthermore, the cable should be sufficiently flexible to traverse the hinges between the laptop screen and base. This study aims to design a cable with a total thickness of less than 0.6 mm and an insertion loss of less than 10 dB/m at a frequency of 6 GHz. Polytetrafluoroethylene (PTFE), a flexible material, can be used to meet these requirements. We simulate the characteristics of various PTFE layer thicknesses. The trend shows a thicker PTFE layer and lower insertion loss. Finally, we fabricate and test two structures with different thicknesses. Both thicknesses are less than 0.6 mm, and the insertion losses are less than 10 dB/m at 6 GHz. We demonstrate the feasibility of the proposed design and fabrication process for these applications through simulations and measurements.

## 1. Introduction

**Figure 1:**
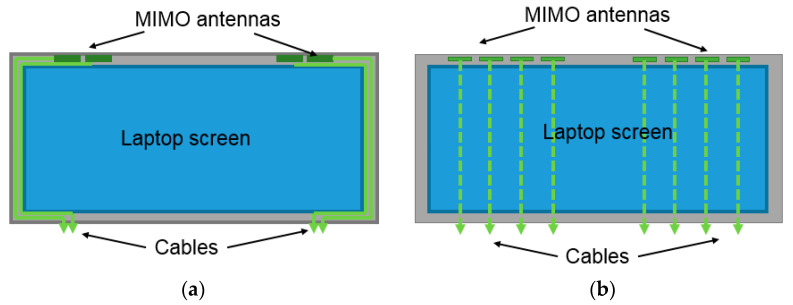
Connection cables placement scenarios on a laptop. (**a**) Cables passing through the bezel. (**b**) Cables under the screen. Scenario (**b**) can have more and shorter cables than scenario (**a**).

In 5G, Wi-Fi 6, and future high-speed communications, the number of antennas installed in a mobile device will increase to obtain a higher data rate and throughput by applying multiple-input, multiple-output (MIMO) technology [1,2,3,4]. MIMO antennas are mounted around the bezel of a laptop display. Generally, the circular mini cables between the MIMO antennas and laptop base are placed around the bezel, as shown in Figure 1a. Narrower bezels and larger display-to-body ratios are the current laptop trends. The space inside the bezel for the placement of circular mini cables is limited. To obtain more space, another cable placement scenario is presented in Figure 1b. In the scenario shown in Figure 1a, the number of MIMO antennas and connection cables is limited by the bezel space. In the scenario shown in Figure 1b, the connection cables are placed under the screen panel; therefore, the number of cables can be greater than in the scenario shown in Figure 1a. The most challenging part of the scenario in Figure 1b is that the connection cables must be sufficiently thin to prevent the water ripple effect on the laptop screen. In addition to the thickness constraints, the scenario shown in Figure 1b is characterized by a shorter connection length (less loss) and more cable placement space (more possible cables) than the scenario shown in Figure 1a.

Thus, several requirements must be satisfied to manufacture a cable to satisfy the scenario shown in Figure 1b. First, the cable should be sufficiently thin to prevent the screen water ripple effect while undertaking the screen panel. A flimsy stripline structure in the microstrip line family is a good candidate to satisfy this requirement [5,6,7,8]. A stripline structure was adopted in this study because it is thin and has a confined electromagnetic (EM) field. Second, the cable should be sufficiently flexible to pass through the hinges between the base and display of the laptop. Polytetrafluoroethylene (PTFE), a material that exhibits high flexibility and low loss, is widely used in many high-frequency applications [9,10,11,12]. Therefore, PTFE was selected as the stripline substrate material.

The stripline structure and PTFE properties are introduced in Section 2. The preliminary study of stripline on stacked PTFE substrates without a bonding layer is described in Section 3. A simulation of the thickness with the bonding layer is presented in Section 4. The experiment and validation results are presented in Section 5.

## 2. Stripline and PTFE

**Figure 2:**
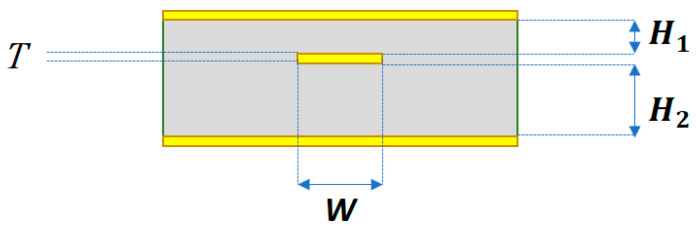
Cross-section of a typical stripline.

The stripline shown in Figure 2 belongs to the microstrip line family and has been extensively studied [5,6,7,8]. In Figure 2, the top and bottom metals serve as the ground layers, and the middle metal serves as a signal line. *T* and *W* are the thickness and width of the signal line, respectively. *H*_1_ is the substrate thickness between the signal line and the top ground metal. *H*_2_ is the substrate thickness between the signal line and the bottom ground metal. The scenario in which *H*_1_ = *H*_2_ is called a symmetric stripline, whereas that in which *H*_1_ is not equal to *H*_2_ is called an asymmetric stripline. Although single-layer microstrip line structures are thinner, they may result in higher EM interference or radiation. The stripline structure has a more confined EM field than a single-layer microstrip structure; therefore, the stripline structure was adopted in this study. Before applying the full-wave EM simulations, either the formula in Refs. [5,6,7] or an online stripline impedance calculator [8] was used to quickly estimate the dimensions of the stripline to fit the targeted characteristic impedance.

**Figure 3:**
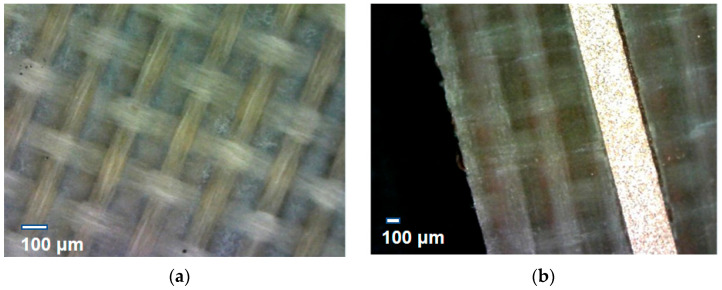
(**a**) PTFE substrate under the microscope. (**b**) Fabricated signal line on the PTFE substrate.

In this study, because the stripline connection may pass through the hinges between the laptop screen and laptop base, the stripline must be flexible. PTFE is both flexible and has excellent physical, chemical, electrical, and heat resistance properties. PTFE is widely used in many high-frequency applications owing to its low-loss property [9,10,11,12]. The PTFE substrate used in this study is shown in Figure 3a. The PTFE substrate with the fabricated signal line is shown in Figure 3b. The signal line was fabricated using lithography. In Figure 3b, the metal width is approximately 0.3 mm. The laminated prepreg (PP) comprised cyclic olefin copolymer (COC). The PP type was 1080. The electrical properties of the PTFE and PP used in this study are summarized in Table 1.

## 3. Preliminary Simulation and Experiment

The target specifications in this study were a total thickness < 0.6 mm, return loss < 15 dB, and insertion loss < 4 dB/m at 1 GHz and <10 dB/m at 6 GHz, as shown in Table 2. The operating frequency ranged from 0 to 6 GHz, which covered communication applications below 6 GHz.

**Figure 4:**
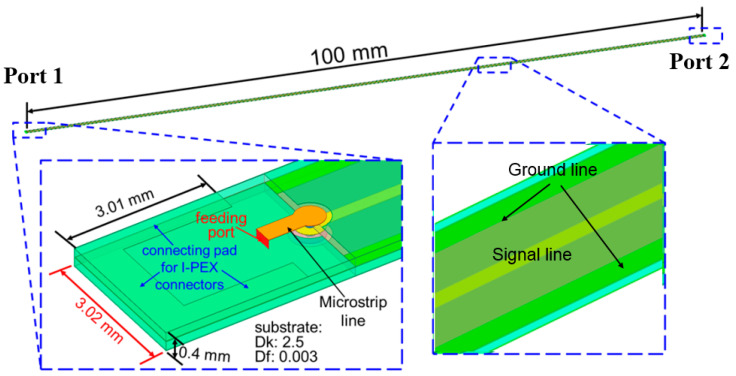
Preliminary simulation setup for full-wave EM simulation software.

**Figure 5:**
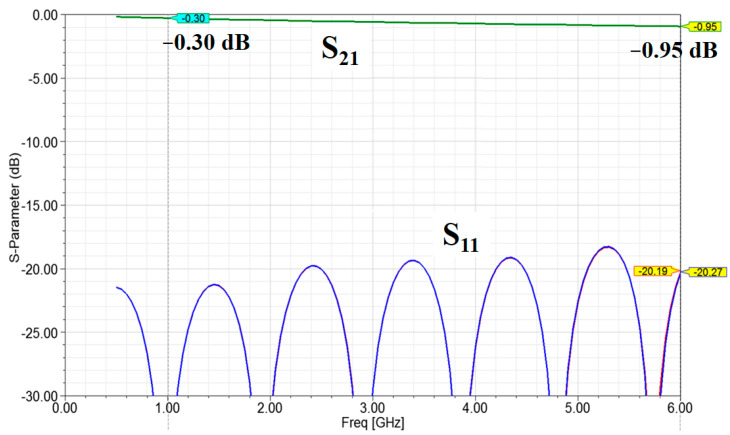
Simulation setup for full-wave EM simulation software. S_11_ is the return loss of port 1. S_21_ is the insertion loss from port 1 to 2.

The preliminary simulation setup for the full-wave EM simulation software (ANSYS HFSS) is shown in Figure 4. Before applying the full-wave EM software, the dimensions of the stripline were estimated using an online calculator [8]. The simulation length was 100 mm. The lower left plot shows the transition part design between the stripline and outside connector. The lower right plot shows the stripline structure with a 0.3 mm wide signal line and two 0.4 mm wide parallel ground lines. These two parallel ground lines were used to provide a more confined EM field in the preliminary study. The total thickness was 0.42 mm, which included two 0.2 mm thick PTFE layers and two 0.1 mm thick metal layers for the top and bottom ground layers. The simulation results are shown in Figure 5. The return loss was <15 dB in the 0–6 GHz operating range. The insertion loss was 0.3 dB per 0.1 m at 1 GHz and 0.95 dB per 0.1 m at 6 GHz. Hence, the simulated insertion loss was 3 dB/m at 1 GHz and 9.5 dB/m at 6 GHz. All the preliminary simulation results satisfied the target specifications.

**Figure 6:**
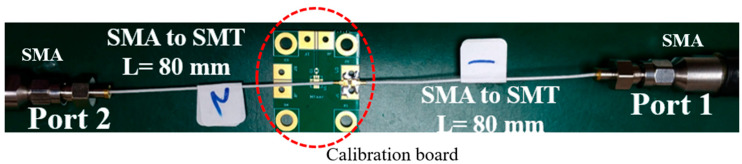
Calibration setup for the loss between SMA and SMT connectors.

**Figure 7:**
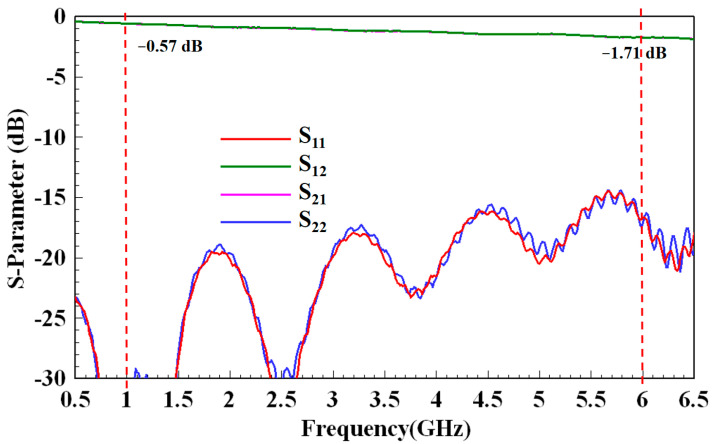
Measurement result of the calibration setup in Figure 5. S_11_ and S_22_ are the return losses of ports 1 and 2, respectively. S_21_ is the insertion loss from port 1 to port 2, and S_12_ is the opposite.

The setup used to calibrate the loss between the SMA and SMT connectors is shown in Figure 6. The calibration board included two SMT connectors, a transition region (as shown in the lower-left part of Figure 4), and a short stripline structure. From the measurement results in Figure 7, the return loss was <15 dB, and the insertion loss was 0.57 dB at 1 GHz and 1.71 dB at 6 GHz. This provided the values to de-embed the losses between the network analyzer and test the stripline samples in the flowing measurements.

**Figure 8:**
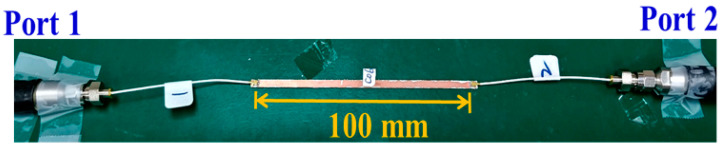
Measurement setup with the test structure and two SMT to SMA cables.

**Figure 9:**
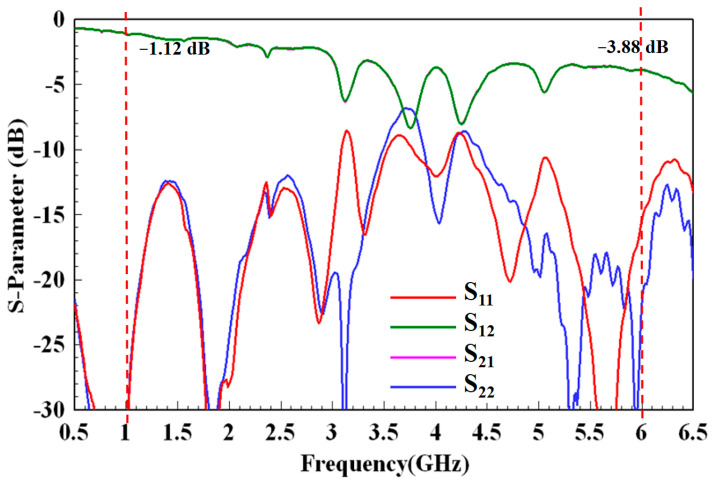
Measurement result of the preliminary test structure.

The overall measurement setup of the test structure and two SMT for the SMA cables is shown in Figure 8. The measurement results for the preliminary test structure are shown in Figure 9. Because the stripline length was 100 mm, the de-embedding insertion loss (dB/m) was ((measured data) − (calibrated loss value)) × 10. From Figure 9, the measured insertion loss was 1.12 dB at 1 GHz; therefore, (1.12 dB − 0.57 dB) × 10 = 5.5 dB/m. Moreover, the measured insertion loss was 3.88 dB at 6 GHz; therefore, (3.88 dB − 1.71 dB) × 10 = 21.7 dB/m. The return loss was greater than 10 dB for several bands. The insertion and return losses were worse than those in the corresponding simulations in Figure 4.

**Figure 10:**
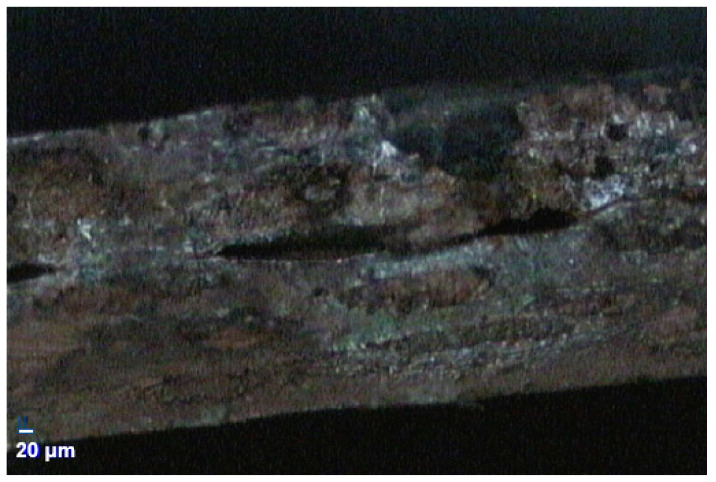
PTFE layers are not properly laminated without a bonding layer.

After further investigation, two parts of the design were modified. First, in the preliminary fabrication process, only two PTFE layers were used to heat up and press together, without bonding material. This is similar to the preliminary simulation setup shown in Figure 4. Without a bonding layer or material, the two PTFE layers were not properly laminated in some portions of the stripline, as shown in Figure 10. A fabrication process using a bonding layer is proposed in the next section. Second, the parallel ground lines, shown in Figure 4, make the cables more rigid and less flexible. This is also discussed and modified in the next section.

## 4. Simulations of the Modified Designs

Several simulations were performed to further explore the designs and optimal solutions.

### 4.1. Vias vs. Ground Line

**Figure 11:**
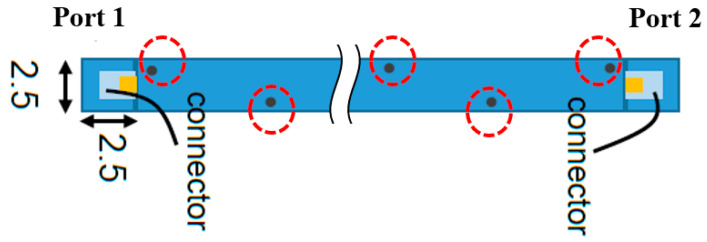
Simulation setup of via placement. The vias are in red dashed circles.

**Figure 12:**
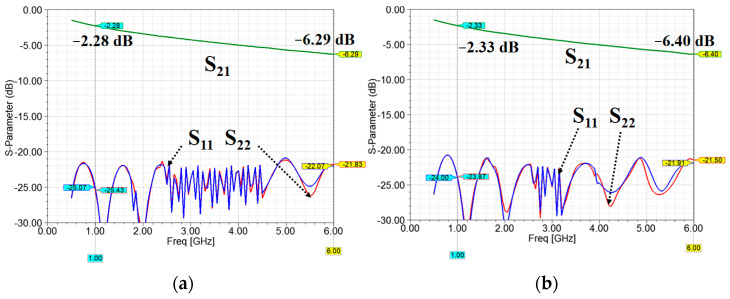
Simulated insertion loss and return loss of (**a**) parallel ground lines, and (**b**) asymmetric via placement.

In the previous section, parallel ground lines were designed to confine the EM field and reduce radiation loss. However, the two parallel ground lines caused the entire stripline to become less flexible. The new stripline structure with vias is shown in Figure 11. The vias were utilized to connect the top and bottom ground metal layers to maintain good ground conduction. The radius of the vias was 0.5 mm. The via placement was asymmetric along the stripline edges. As observed, asymmetric via placement was more flexible than symmetric via placement. The distance between two vias along the same edge was 5 mm. The simulation results for the parallel ground lines and vias are shown in Figure 12, according to which the return losses were all <20 dB in the two scenarios. The insertion loss of the parallel ground line scenario was 2.28 dB at 1 GHz and 6.29 dB at 6 GHz. The insertion loss of the asymmetric via placement scenario was 2.33 dB at 1 GHz and 6.40 dB at 6 GHz. From the simulations, the characteristics of the two scenarios were found to be similar. Thus, the use of asymmetric via placement is feasible.

### 4.2. Insertion Loss vs. PTFE Thickness

**Figure 13:**
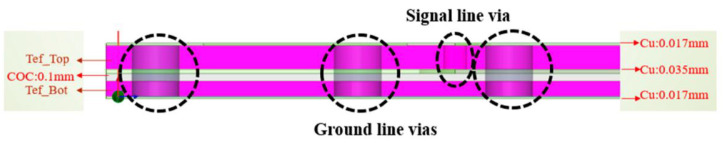
Simulation setup for insertion loss investigation.

Several simulations were performed to explore the characteristics of PTFE thicknesses. The simulation setup for insertion loss investigation is shown in Figure 13; this represents the side view of a stripline. Ground line vias with a 0.5 mm radius connected the top and bottom metal layers. Signal line vias with a 0.3 mm radius connected the SMT connector and signal line layers. The thickness of the top and bottom layers was 0.017 mm. The thickness of the signal line layer was 0.035 mm. The thickness of the bonding layer (COC) was 0.1 mm.

**Figure 14:**
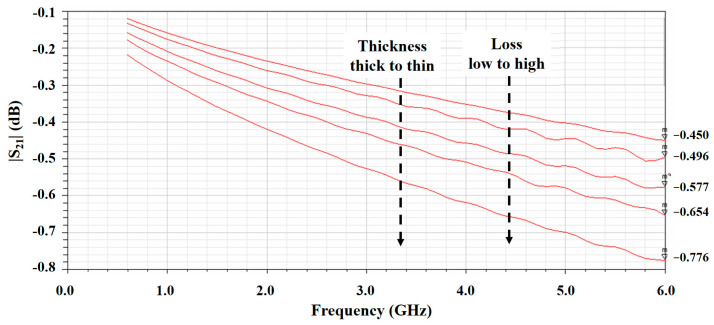
Simulation results of total thickness investigations.

The five thickness scenarios are presented in Table 3. The total thickness included the thicknesses of the top and bottom metal layers, as well as of the bonding layer. Each corresponding signal linewidth was tuned to maintain the return loss below 20 dB in the operation band. The simulated insertion losses for different total thickness scenarios are summarized in Table 3 and Figure 14. Again, the length of the stripline was 100 mm. Therefore, the insertion losses at 6 GHz in Figure 14 was multiplied by 10 to obtain the insertion loss (dB/m) presented in Table 3. As observed from the simulation trends, the thicker the PTFE layer, the lower the insertion loss. Furthermore, from the simulation results of insertion loss at 6 GHz, the total thickness might be thinner than 0.5 mm and still meets the insertion loss of <10 dB/m at the 6 GHz target.

## 5. Experiments

**Figure 15:**
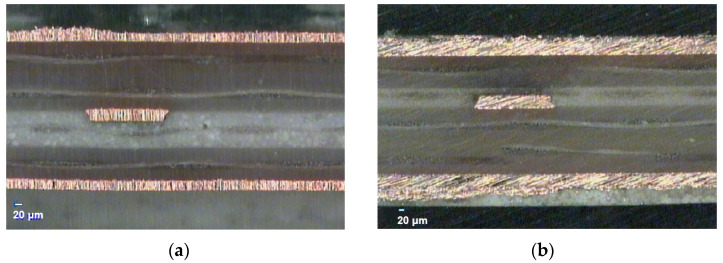
Cross-section of the fabricated striplines in (**a**) scenario 2 and (**b**) scenario 4.

The insertion losses of the stripline structure with the PP between PTFE layers could be logically determined from the results of the simulations in Section 4. The insertion losses of the asymmetric via placement are also feasible. In the experiment, the stripline dimensions of Scenarios 2 and 4 listed in Table 3 were selected for fabrication. The cross-sections of the fabricated striplines are shown in Figure 15. The simulated and measured total thicknesses of the structure in Scenario 2 were 0.588 mm and 0.594 mm, respectively; those in Scenario 4 were 0.515 mm and 0.520 mm, respectively.

**Figure 16:**
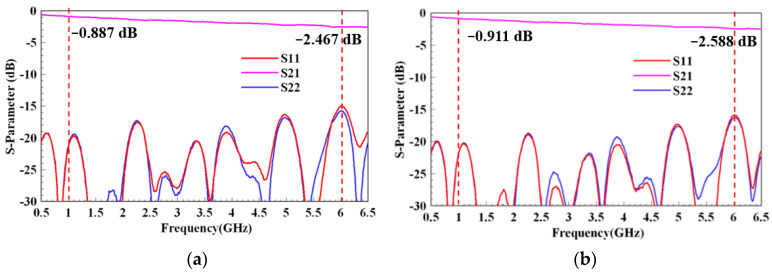
Measurement results of (**a**) Scenario 2 and (**b**) Scenario 4.

The measurement results for Scenarios 2 and 4 are shown in Figure 16. A comparison of the simulation and measurement data is presented in Table 4. Notably, the measured insertion loss followed the de-embedding process described in Section 3 to obtain the de-embedding insertion loss data provided in Table 4. After modifying the fabrication process with the bonding layer and via placement, the PTFE layers were properly laminated, and the electrical characteristics also followed the simulation trend; that is, the thicker the PTFE layer, the lower the insertion loss. Table 4 and Figure 16 show that the measured data also satisfied the target specifications and were close to the simulation results. The thicknesses of the two scenarios were less than 0.6 mm. The return losses were greater than 15 dB in both scenarios. The insertion losses of the two scenarios were less than 4 dB/m at 1 GHz and less than 10 dB/m at 6 GHz. This proposed stripline design and fabrication process show potential for use in high-speed applications such as laptop MIMO antenna connections.

## 6. Conclusions

This study investigated the PTFE fabrication process and the stripline design. The application of this design is aimed at sub-6 GHz communications. The thickness of stripline structures was limited to 0.6 mm to satisfy the needs of laptop applications. The characteristics of the different stripline thicknesses were simulated under this requirement. Two thickness scenarios were selected for fabrication. The measurement data met the target specifications and matched the simulation results. The proposed flexible thin flimsy stripline design and fabrication process was proven feasible for low-loss and high-speed applications through simulations and measurements.

## Figures and Tables

**Table 1:** Electrical properties of PTFE and PP.

	Dielectric Constant at 10 GHz (Dk)	Dissipation Factor at 10 GHz (Df)
**PTFE**	2.2	0.0009
**PP (COC)**	2.9	0.0018

**Table 2:** Target specifications.

**Operating frequency**	0–6 GHz
**Total thickness**	<0.6 mm
**Return loss**	<15 dB
**Insertion loss**	<4 dB/m @ 1 GHz <10 dB/m @ 6 GHz

**Table 3:** Simulation scenarios of different PTFE thicknesses.

	Thickness of Top PTFE Layer (mm)	Thickness of Bottom PTFE Layer (mm)	Total Thickness (mm)	Signal Line Width (mm)	Insertion Loss at 6 GHz (dB/m)
**Scenario 1**	0.254	0.254	0.642	0.39	4.50
**Scenario 2**	0.254	0.2	0.588	0.34	4.96
**Scenario 3**	0.2	0.2	0.534	0.31	5.77
**Scenario 4**	0.254	0.127	0.515	0.27	6.54
**Scenario 5**	0.2	0.127	0.461	0.25	7.76

**Table 4:** Summary of specification, simulation, and measurement data.

		Specification	Simulation		Measurement	
			Scenario 2	Scenario 4	Scenario 2	Scenario 4
Operating frequency (GHz)		0~6	0~6	0~6	0~6	0~6
Total thickness (mm)		<0.6	0.588	0.515	0.594	0.520
Return loss (dB)		<15	<15	<15	<15	<15
Insertion loss (dB/m)	@ 1 GHz	<4	1.82	2.41	3.17	3.41
	@ 6 GHz	<10	4.96	6.54	7.57	8.78

## Data Availability

The data presented in this study are available on request from the corresponding author. The data are not publicly available as the data also form part of an ongoing study.
